# Development of PCR Blocking Primers Enabling DNA Metabarcoding Analysis of Dietary Composition in Hematophagous Sea Lamprey

**DOI:** 10.1002/ece3.71999

**Published:** 2025-08-20

**Authors:** Conor O'Kane, Nicholas S. Johnson, Kim T. Scribner, Jeannette Kanefsky, Weiming Li, John D. Robinson

**Affiliations:** ^1^ Department of Fisheries and Wildlife Michigan State University East Lansing Michigan USA; ^2^ United States Geological Survey, Great Lakes Science Center Hammond Bay Biological Station Millersburg Michigan USA

**Keywords:** blocking primers, DNA metabarcoding, metagenomics, quantitative PCR, sea lamprey12S

## Abstract

Conventional dietary assessments are challenging in hematophagous species, particularly in sea lamprey ( *Petromyzon marinus* ). However, recent technological developments and molecular approaches have provided an attractive alternative through the use of DNA metabarcoding. While DNA metabarcoding has been used for dietary analyses in numerous species, including lampreys, applications of universal primers that detect a diverse set of prey items can be limited by the amplification of predator DNA. In this study, we designed and tested eight blocking primers designed to suppress the amplification of sea lamprey DNA with vertebrate‐universal primers targeting the mitochondrial 12S rRNA gene. This approach allowed for the use of a single marker to amplify a taxonomically diverse suite of host species, in contrast to previous studies that used multiple taxon‐specific primer pairs (e.g., Salmonidae, Cyprinidae, and Catostomidae). Candidate blocking primers evaluated in this study differed in base pair length, end sequence modification, and purification method. Samples with different sea lamprey‐to‐host DNA ratios were subjected to multiple detection methods including gel electrophoresis, quantitative PCR, and DNA metabarcoding to assess the ability of each blocking primer to selectively suppress amplification of the sea lamprey 12S gene region. All blocking primers tested performed well and demonstrated high effectiveness, suppressing sea lamprey reads by > 99.9% in mock communities and improving host DNA sequence recovery across various sample types, including wild‐caught lamprey. Results show that the blocking primers evaluated can facilitate molecular diet analysis in sea lamprey, allowing the amplification of a taxonomically diverse range of host fish species with universal primers.

## Graphical abstract

Eight blocking primers were developed and tested to reduce sea lamprey DNA amplification when using universal 12S rRNA primers for dietary analysis via DNA metabarcoding. These primers effectively suppressed sea lamprey DNA, allowing for clearer detections of host species within digestive tract samples. This study provides a framework for detailed investigations into blocking primer effectiveness while improving lamprey dietary studies by allowing the use of universal primers in place of multiple taxon‐specific primers.

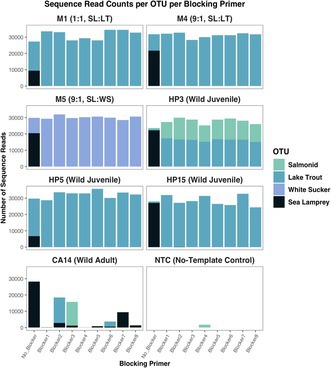

## 1 Introduction

The invasion of parasitic sea lamprey ( *Petromyzon marinus* ) into the Great Lakes during the mid‐20th century and resulting impacts to both native fish communities and sport and commercial fishing activities required international management efforts (Coble et al. 1990; Lawrie 1970). Following initial invasion and establishment, sea lamprey attacks contributed to the loss of over 95% of the lake trout ( *Salvelinus namaycush* ) stock biomass and precipitous numerical declines in lake whitefish ( *Coregonus clupeaformis* ), walleye ( *Sander vitreus* ), and other economically and ecologically valuable fisheries (Smith and Tibbles 1980). In response, the Great Lakes Fishery Commission (GLFC) was established in 1954 and tasked with implementing control programs designed to reduce sea lamprey abundance within the Great Lakes (Meyer and Schnick 1983; Robinson et al. 2021). Control efforts were largely successful and have reduced population levels in the Great Lakes to ~10% of peak abundance (Heinrich et al. 2003; Robinson et al. 2021).

Uncertainty remains about the damage that sea lamprey cause to spatially and taxonomically diverse Great Lakes fisheries. Sea lamprey in the Great Lakes have been documented feeding on a variety of species including lake trout, burbot ( *Lota lota* ), suckers (*Catostomus* spp.), pikes (*Esox* spp.), and lake whitefish (Farmer 1980). However, given that sea lamprey are hematophagous and feed mostly on the blood of their prey (Renaud et al. 2009), conventional dietary assessment methods are not applicable. Physical components of the diet, such as bones, shells, and other hard structures, are not present in sea lamprey digestive systems, requiring sea lamprey damage assessments to rely on quantification of wounding or marking rates (Ebener et al. 2003; King Jr. 1980). However, interpretations of marking data are limited, as the focus of current protocols is largely limited to lake trout, rather than all members of the Great Lakes fish community (Firkus et al. 2021). Furthermore, captures of juvenile sea lamprey are often the result of bycatch where anglers or vessels were targeting the lamprey's host fish (typically lake trout), caught either with a lamprey still attached or visible wound markings. As such, samples for dietary analysis are not derived from randomly selected sea lamprey within the lake; rather, they are gathered from captured host fish. Biochemical methods such as stable isotope analysis and fatty acid profiles have been previously used to circumvent these capture biases in wounding data assessments (Happel et al. 2017; Harvey et al. 2008). However, while these methods are helpful in gathering larger ecological insights such as trophic level placement, they are unable to comprehensively and compositionally characterize sea lamprey diets at the species level.

Recent advances in molecular diet analysis offer an attractive alternative that addresses these shortcomings and limitations (Pompanon et al. 2012). While various electrophoretic techniques have been used for dietary analyses previously (Deagle et al. 2005; Symondson 2002; Walrant and Loreau 1995), the rise of next‐generation sequencing more recently has led to widespread accessibility of DNA metabarcoding for dietary assessments. As reviewed in Pompanon et al. (2012), DNA metabarcoding allows for a species‐specific prey designation via PCR amplicons sequenced from host DNA extracted from gut contents. Primers specific to certain taxonomically conserved gene regions, such as the mitochondrial 12S and 16S ribosomal RNA genes (Deagle et al. 2009; Riaz et al. 2011, respectively), allow for the amplification of sequences from a wide variety of taxa. Amplified products can be subsequently aligned to databases of known sequences to provide reliable taxonomic classifications (Yang et al. 2014). This method has been widely applied for dietary studies on mammals (Berry et al. 2017; Buglione et al. 2018; Lopes et al. 2020), birds (Hacker et al. 2021; McClenaghan et al. 2019), fishes (Berry et al. 2017; Harms‐Tuohy et al. 2016; Jakubavičiūtė et al. 2017), and other taxa, including the flesh‐feeding Arctic lamprey ( *Lethenteron camtschaticum* ; Shink et al. 2019) and sea lamprey in the Great Lakes (Johnson et al. 2021).

Johnson et al. (2021) introduced a method for identifying host species using DNA extracted from sea lamprey feces using three taxon‐specific primers that individually targeted salmonids, catostomids, and cyprinids. Their results showed that diet composition varied between sea lamprey captured in the northern basin of Lake Huron and those from a tributary of Lake Huron. This study provided empirical evidence supporting the concept and feasibility of DNA barcoding in lamprey dietary analyses, but its use of multiple taxon‐specific primers limited their ability to compare relative sequence abundance (Hänfling et al. 2016) of multiple hosts detected in individual sea lamprey fecal samples (Johnson et al. 2021). While beneficial for detecting a wider range of hosts, use of more conserved vertebrate primers also amplifies large amounts of sea lamprey DNA, lowering the proportion of usable data. Reductions decrease the effectiveness of higher data outputs in mitigating sequence read biases introduced by extraction and amplification stochasticity (Alberdi et al. 2018; Leray and Knowlton 2017; Polz and Cavanaugh 1998).

One method of counteracting the amplification of sea lamprey DNA is to apply a blocking primer (Vestheim and Jarman 2008). The inclusion of an effective blocking primer during the PCR process can significantly suppress predator DNA amplification while increasing the relative amount of amplified prey fragments in dietary studies (Vestheim et al. 2011). Blocking primers are also capable of blocking amplification of prey sequences, potentially limiting benefits (Piñol et al. 2015). Unlike universal primers, which are designed to anneal broadly to various taxonomic groups, blocking primers are designed to anneal to a specific target. Amplification prevention then occurs either via (1) annealing inhibition, where the blocking primer binding site overlaps with the universal primer binding site and the physical presence of the blocker prevents annealing, or (2) elongation arrest, where the blocking primer attaches downstream and physically prevents nontarget sequence elongation (Vestheim et al. 2011). Once attached to their specific target, the physical presence of the blocking primer prevents amplification of the sequence during PCR. This method has been successfully applied in other DNA metabarcoding diet studies (Jakubavičiūtė et al. 2017; Leray et al. 2013; Su et al. 2018). To further enhance the performance of blocking primers, end modifications that decrease the possibility of amplification from the blocking primer itself, such as a 3′ C3 spacer (Homma et al. 2022; Nelson et al. 2017; Robeson II et al. 2018) or a 3′ inverted dT (Egizi et al. 2013), have been implemented in other dietary studies. The use of a blocking primer was not necessary in Johnson et al. (2021) as they used taxon‐specific primers. However, a universal primer, in this case, would be more efficient as it could detect multiple families with fewer PCR amplifications, along with unsuspected prey items. A successful sea lamprey blocking primer in this context would facilitate dietary studies in sea lamprey as it would allow for the use of universal primers, thus providing a more complete characterization of the diet in a single amplification reaction.

This study focused on the design and testing of blocking primers to determine their effectiveness for amplification suppression of the sea lamprey 12S rRNA gene region, while allowing amplification of host species DNA. An annealing inhibiting blocking primer design was selected over an elongation arrest blocker given its expected higher efficiency (Vestheim et al. 2011). We designed eight blocking primers, representing each possible combination of three primer design features: base pair (bp) length, end sequence modification, and purification method. We then tested blocking primer effectiveness using three evaluation methods (visualization of conventional PCR amplification products, quantitative PCR, and DNA metabarcoding), each applied to single and mixed‐species DNA templates in mock communities with known concentrations and dietary samples of wild‐caught sea lamprey.

## 2 Methods

### 2.1 Blocking Primer Development

Sequences of the mitochondrial 12S rRNA gene region from 149 Great Lakes fish species (*n* = 213 total sequences, ranging in length from 89 to 107 bp) were obtained from NCBI's GenBank database (Sayers et al. 2022). Multisequence alignments were created using MEGA (v. 6.0; Tamura et al. 2013) for comparison of the sequences to the same gene region in sea lamprey. Additionally, the forward 12S‐V5 primer set (Riaz et al. 2011) sequence was appended to the 5′ end of the sequence alignment to facilitate the design of an annealing inhibiting blocking primer.

**FIGURE 1:**
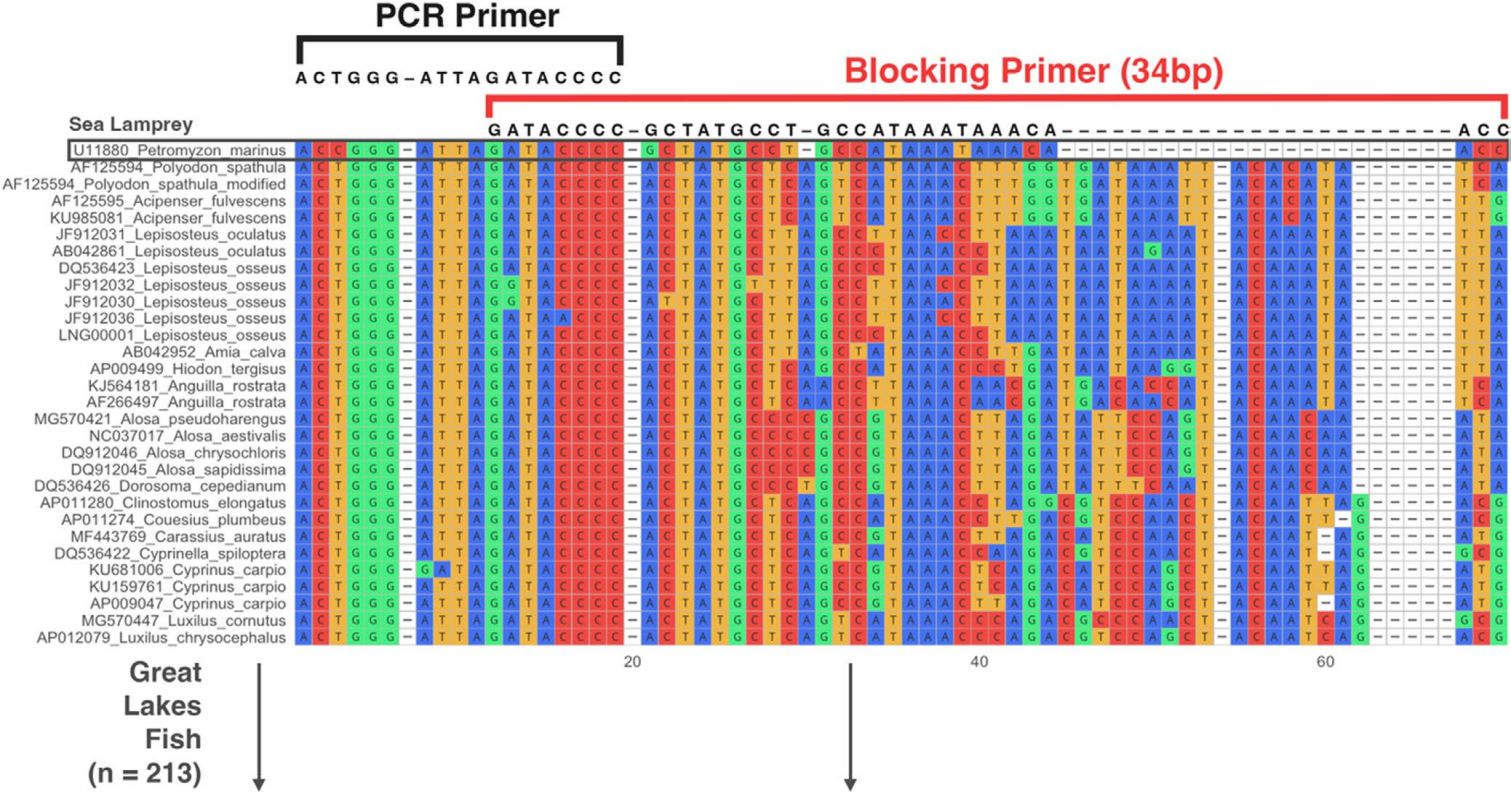
Visualization for the overlap of a 34 bp blocking primer with the universal 12S PCR primer. The first sequence is the target sequence (sea lamprey), while the following sequences represent the first 30 sequences of Great Lakes fish from the constructed sequence database of 213 sequences. The first 75 nucleotides of the alignment are shown, with the total length being 156 nucleotides (including gaps and primers).

Variation in the sequence length can affect primer species specificity and annealing temperature (Vestheim et al. 2011). For this study, primer lengths of 34 bp and 36 bp were selected to include a 23 bp gap (Figure 1) that was noted in the alignment between sea lamprey and other Great Lakes fishes. Targeting this gap was expected to enhance primer specificity for sea lamprey sequences relative to prey fish DNA sequences.

Two primer end modifications were compared to characterize blocking primer effectiveness: a C3 spacer (three hydrocarbons added at the 3′ end) and a 3′ inverted dT (reverse‐linked nucleotide). Both additions are standard with most oligonucleotide suppliers and may improve blocking primer efficiency by inhibiting both DNA polymerase extension and 3′ exonuclease degradation (Egizi et al. 2013; Liu et al. 2019). For our study, four of the eight blocking primers tested contained a C3 spacer, while the other four contained an inverted dT modification.

We also included different purification methods for the blocking primers tested in this study. Desalting is typically used by most vendors, while high‐performance liquid chromatography (HPLC) purification is recommended for usage with blocking primers for superior binding efficiency (Vestheim et al. 2011). Eight blocking primers were developed, representing all combinations of these variables (purification method, end modification, and sequence length; Table 1). The eight blocking primers evaluated in this study were synthesized and purified by Integrated DNA Technologies (Coralville, IA).

**TABLE 1:** Table of the eight blocking primers designed and tested in this study.

Blocking primer	Purification method	Length (bp)	End modification	Sequence (5′–3′)	Tm^a^
Blocker 1	HPLC	34	C3 spacer	GATACCCCGCTATGCCTGCCATAAATAAACAACC/3SpC3/	63.8
Blocker 2	HPLC	36	C3 spacer	GATACCCCGCTATGCCTGCCATAAATAAACAACCGT/3SpC3/	65.8
Blocker 3	HPLC	34	Inverted dT	GATACCCCGCTATGCCTGCCATAAATAAACAACC/3InvdT/	63.8
Blocker 4	HPLC	36	Inverted dT	GATACCCCGCTATGCCTGCCATAAATAAACAACCGT/3InvdT/	65.8
Blocker 5	Desalted	36	Inverted dT	GATACCCCGCTATGCCTGCCATAAATAAACAACCGT/3InvdT/	65.8
Blocker 6	Desalted	36	C3 spacer	GATACCCCGCTATGCCTGCCATAAATAAACAACCGT/3SpC3/	65.8
Blocker 7	Desalted	34	Inverted dT	GATACCCCGCTATGCCTGCCATAAATAAACAACC/3InvdT/	63.8
Blocker 8	Desalted	34	C3 spacer	GATACCCCGCTATGCCTGCCATAAATAAACAACC/3SpC3/	63.8

*Note:* The primer name, length (bp), end modification, purification method, 5′‐3′ sequence, and melting temperature (*T*_*m*_) for each is given.

^a^ Tm calculated using nearest neighbor method.

### 2.2 Primer Evaluation

#### 2.2.1 Conventional PCR

An initial assessment of blocking primer effectiveness was conducted using conventional PCR and 1% agarose gel (General Purpose Agarose GP2; Midwest Scientific, Fenton, MO) electrophoresis to visualize amplified products. Single‐species DNA samples from multiple individual sea lamprey and two Great Lakes native host fish species (lake trout and walleye) were diluted to 5 ng/μL prior to amplification (extractions followed protocol from DNeasy Blood and Tissue kit; Qiagen, Hilden, Germany). Lake trout was selected due to the current understanding that the species constitutes a large component of sea lamprey diets (Lantry et al. 2015). Walleye was selected given its potential as a host and higher sequence similarity to sea lamprey within the region of interest (see Data Accessibility Statement). Each of the eight blocking primers was evaluated in all six single‐species DNA samples for 48 total samples that included both a blocking primer and DNA from either sea lamprey, lake trout, or walleye. For each of the six single‐species samples, a no‐blocking primer sample was included as a positive amplification control. Additionally, each blocking primer was included in a no DNA, PCR negative amplification control. Blocking primers were tested at a relative concentration of 10:1 to unmodified 12S primers (12S‐V5; Riaz et al. 2011), following recommendations from Vestheim et al. (2011).

PCR conditions were modified from those used in a previous eDNA metabarcoding study (Pukk et al. 2021). Briefly, PCR was performed in a 15 μL reaction volume with 1.5 μL of 10X AmpliTaq Gold PCR Buffer II (Applied Biosystems, Waltham, MA), 0.36 μL of dNTPs (10 mM), 1.2 μL of MgCl_2_ (25 mM), 0.75 μL of BSA (20 mg/mL), 0.8 μL of unmodified 12S F and R primers (10 μM) and blocking primers (100 μM), 6.54 μL of Millipore water (UV treated), 0.25 μL of AmpliTaq Gold (5 U/μL), and 2 μL of template DNA (20 ng/μL) for all species. Positive control samples replaced blocking primer additions with Millipore water. Negative control samples replaced DNA template with water. Thermal conditions for PCR were as follows: 10 min at 95°C (1×); 30 s at 95°C, 30 s at 57°C, 45 s at 72°C (40×); and 5 min at 72°C. Following amplification, 4 μL of PCR product and 2.5 μL of glycerol loading dye were run on a 1% agarose gel with GelRed stain (0.5×; Biotium, Fremont, CA). Gels were photographed under UV light in a Labnet Enduro GDS II imaging system (Labnet HQ, Edison, NJ).

#### 2.2.2 Quantitative PCR

To characterize PCR amplification efficiency of mixed‐species samples, three mock communities were set up using varying DNA concentration ratios of sea lamprey and either lake trout or white sucker (community combinations M1, M4, and M5; Table 2). Alongside lake trout, white sucker is a known host fish of sea lamprey in the Great Lakes (Johnson et al. 2021) and was thus included in this analysis. All DNA samples of sea lamprey and host species were diluted to 5 ng/μL prior to creating the multispecies mixture (mock community) using a NanoDrop Spectrophotometer (Thermo Fisher Scientific, Waltham, MA) for quantification. M1 was an equal ratio of sea lamprey DNA and lake trout DNA (1:1) assembled from samples of known DNA concentrations. M4 and M5 contained skewed 9:1 ratios of sea lamprey DNA to lake trout and white sucker DNA, respectively. Testing both uniform and skewed lamprey‐to‐host DNA ratios allowed for the comparisons of DNA amplification at equal concentrations, along with comparisons in samples that more closely align with digestive tract samples from wild‐caught sea lamprey in preliminary analyses (where sea lamprey sequences comprised ~90% of all sequence reads; unpublished data). Additionally, single‐species DNA samples were again used, with two replicates of sea lamprey, lake trout, walleye, and white sucker incorporated into analyses. All single‐species samples were diluted to 5 ng/μL for consistency with the mixed‐species samples.

**TABLE 2:** Table of DNA samples used across testing methods within this study, showing sample name, species DNA included in the sample, and details of which individual for single‐species samples, lamprey‐to‐host DNA concentration ratios for mixed‐species samples, or capture data for the wild‐caught samples.

Sample	Species	Details	Evaluation method
SL1	Sea Lamprey	Individual 1	Gel, qPCR
SL2	Sea Lamprey	Individual 2	Gel, qPCR
LT1	Lake Trout	Individual 1	Gel, qPCR
LT2	Lake Trout	Individual 2	Gel, qPCR
WAE1	Walleye	Individual 1	Gel, qPCR
WAE2	Walleye	Individual 2	Gel, qPCR
WS1	White Sucker	Individual 1	qPCR
WS2	White Sucker	Individual 2	qPCR
M1	Sea Lamprey, Lake Trout	50:50 Lamprey:Host	qPCR, Metabarcoding
M4	Sea Lamprey, Lake Trout	90:10 Lamprey:Host	qPCR, Metabarcoding
M5	Sea Lamprey, White Sucker	90:10 Lamprey:Host	qPCR, Metabarcoding
HP3	Wild‐Caught (Mixed)	Huron (juvenile)	qPCR, Metabarcoding
HP5	Wild‐Caught (Mixed)	Huron (juvenile)	Metabarcoding
HP15	Wild‐Caught (Mixed)	Huron (juvenile)	Metabarcoding
CA14	Wild‐Caught (Mixed)	Champlain (adult)	Metabarcoding

The digestive contents of a wild‐caught sample, HP3 (Huron Parasitic 3), were also included in qPCR‐based evaluations. For this sample and all other wild‐caught samples in this study, juvenile sea lamprey were frozen whole following collection from either Lake Huron or Lake Champlain and sent to Hammond Bay Biological Station, where they were stored at −80°C for further analysis. Sea lamprey digestive tracts were removed, and DNA extractions followed the protocol used by Johnson et al. (2021) with the gMax Mini Genomic DNA Kit (IBI Scientific, Dubuque, IA). Juvenile and adult lamprey were collected by collaborators from Fisheries and Oceans Canada (Lake Huron samples) and the US Fish and Wildlife Service (Lake Champlain sample) under associated collection permits covering sea lamprey control and assessment research.

All samples from Table 2 except for HP5, HP15, and CA14 were subjected to quantitative (q)PCR with each blocking primer and in a no‐blocking‐primer control. A no‐DNA control sample was also analyzed for each blocking primer. qPCR was performed in a 20 μL reaction volume including 10 μL of 2X Forget‐Me‐Not EvaGreen Master mix (low ROX; Biotium, Fremont, CA), 0.8 μL of forward and reverse primers at 10 μM and blocking primers at 100 μM (10× the concentration of 12S primers), 5.6 μL of sterile water, and 2 μL of template DNA. A no‐template control (NTC) was included through the addition of sterile water in place of template DNA. Thermal cycling took place on a QuantStudio 6 (Applied Biosystems, Waltham, MA) and followed the Forget‐Me‐Not EvaGreen Master mix three‐step fast cycling protocol (Biotium, Fremont, CA). Specifically, thermal conditions were as follows: 2 min at 95°C (1×); 5 s at 95°C, 10 s at 57°C, and 20 s at 72°C (40×; imaging at extension step). Amplification plots were used to determine cycle threshold (Ct) values, which were used to assess differences in 12S amplification among samples with and without blocking primers. The degree of suppression was then calculated by assuming a doubling of DNA per cycle and using the difference in cycle thresholds as an exponent of two. Amplified products were also subjected to melt curve analyses immediately following the completion of the final cycle to compare melting temperature (*Tm*) peaks of products from single‐species samples and mixed‐species samples with and without blocking primers.

#### 2.2.3 DNA Metabarcoding

Mixed‐species samples were also subjected to DNA metabarcoding tests to provide species‐specific sequence read counts. Mock community samples M1, M4, and M5 from the qPCR analyses, along with gut contents from three juvenile sea lamprey from Lake Huron (HP3, HP5, and HP15) and an adult sea lamprey captured in a trap during spawning migration from Lake Champlain (CA14) were included in metabarcoding tests (Table 2). Reaction volumes were 15 μL with the same reagent concentrations as the conventional PCR analyses, again substituting UV‐treated Millipore water in place of DNA template for NTC samples and including no‐blocking‐primer controls for all DNA samples.

PCR followed the same conditions as the conventional PCR analysis, and an additional set of samples was run with 25 cycles instead of 40 cycles. This test assessed whether reducing the number of PCR cycles would prevent amplification of sea lamprey sequences in later cycles, as a higher PCR cycle number should increase overall ratios between target and nontarget DNA (Vestheim et al. 2011). Of the eight blocking primers used for the 40‐cycle qPCR tests, two were selected (Blockers 2 and 6) along with an NTC for the additional 25‐cycle run. This selection was made based on the high performance of these two blocking primers in conventional PCR and qPCR analyses. Relative sequence read counts for all members of mock communities and wild‐caught samples were evaluated for both cycle numbers.

A secondary PCR reaction was conducted (after amplification of the 12S gene region) to individually barcode samples for demultiplexing of sequence reads to their corresponding samples. Barcoding reactions included i7 (1 μL; 10 μM) and i5 (2 μL; 5 μM) index primers along with 2X Qiagen Plus MM (5 μL; #206152) and PCR conditions were as follows: 15 min at 95°C (1×); 10 s at 95°C, 30 s at 65°C, 30 s at 72°C (10×); and 5 min at 72°C. PCR products were then pooled and bead size selected at 0.5× and 1.2× bead concentrations to remove long and short sequences, respectively. Pooled libraries were diluted and analyzed via TapeStation automated electrophoresis (High Sensitivity D1000 ScreenTape assay) for sequence length confirmation before being sequenced on a 300‐cycle Illumina MiSeq lane (v2 Micro; 2 × 150 bp paired end) at Michigan State University's Research Technology Support Facility.

Processing of sequencing data into relevant feature tables was performed using the mothur software package (version 1.48.0; Schloss et al. 2009). Sequence reads were demultiplexed, assembled into contigs with the allowance of two mismatches from overlapping paired‐end reads, and subsequently trimmed of primer sequences. Trimming was specified to retain only the overlapping regions.

Following the creation of contigs, sequences were summarized and subsequently screened to filter out reads exceeding 107 bp or large homopolymers. Unique sequences were generated and counted before being aligned to the reference database, then preclustered (0 differences allowed within clusters). Chimeric reads were identified and removed. Classifications were defined for the sequences with a confidence score cutoff of 80% or higher. A pairwise distance matrix was calculated using a similarity threshold of 97% (distance cutoff of 0.03), and sequences were subsequently clustered into operational taxonomic units (OTUs) based upon a 99% sequence similarity threshold using our 12S rDNA Great Lakes fish database (see Data Accessibility Statement). Sequence outputs were then cleaned and organized into a community matrix table with counts by OTU (see Data Accessibility Statement) for subsequent data analyses.

A series of paired *t*‐tests were used to statistically compare the effectiveness of each blocking primer based upon sea lamprey and host fish sequence read ratios. Sea lamprey read counts across the seven dietary samples for each blocking primer were compared to sea lamprey read counts of unblocked samples. Comparisons provided a basis for determining whether mean read counts of sea lamprey DNA decreased with the inclusion of individual blocking primers. Similarly, lake trout read counts across dietary samples with each blocker were compared to lake trout read counts in unblocked samples. For lake trout sequence read comparisons, samples M5 and CA14 were not included, as the M5 sample focused on white sucker as host DNA and CA14 was an outlier wild‐caught sample with very low amounts of host DNA. To correct for multiple tests, a sequential Bonferroni correction was used for both the sea lamprey and lake trout read count comparisons (Holm 1979). Blocking primers that significantly reduced sea lamprey sequence reads while not significantly reducing host fish sequence reads were deemed effective.

## 3 Results

### 3.1 Gel Electrophoresis

**FIGURE 2:**
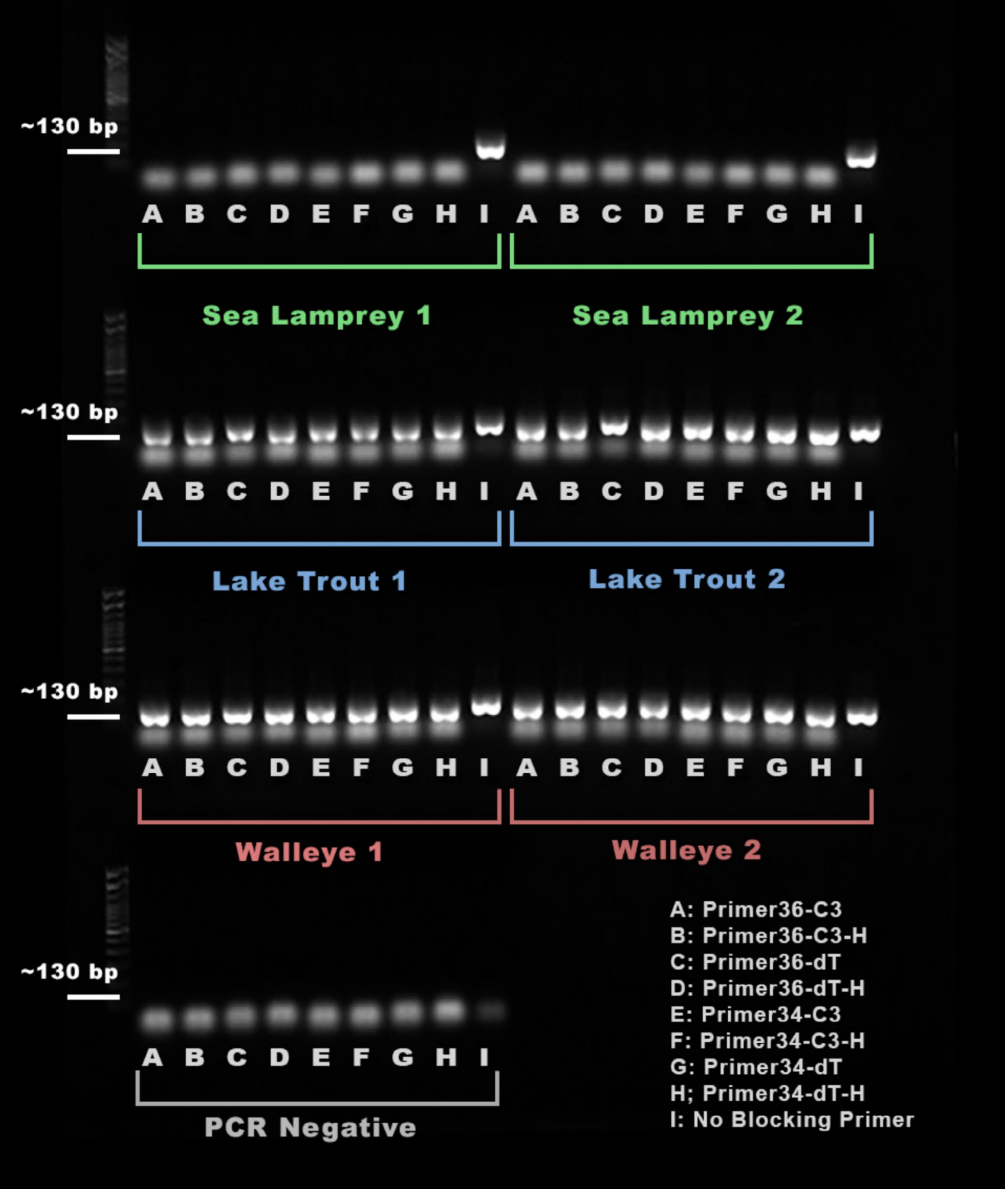
Visualization of results for gel electrophoresis primer evaluation method for both replicates of sea lamprey, lake trout, and walleye. A‐H indicate which primer was included in the sample, and I indicates the sample without a blocking primer. PCR negatives contained no DNA.

PCR amplicon band presence was used to identify successful sample amplification (Figure 2). For sea lamprey, no samples that included a blocking primer showed a visible band of the expected size, while both control samples without a blocking primer showed a band. Results indicated that amplification of sea lamprey DNA was suppressed in all blocking primer samples. For walleye and lake trout replicates, all samples both with and without blocking primers showed a band, indicating no visually identifiable inhibition of host species amplification. The negative controls showed no bands of the expected fragment size in any sample.

While target DNA bands were easily visible, the presence of primer dimers was also noted in all samples that included a blocking primer, including negative controls (Figure 2). Bands well below the target length were considered primer dimers (typically between 10 and 20 bp), as similar results have been found in previous blocking primer analyses (Liu et al. 2019). The presence of these primer dimers prompted the use of a Sage Science BluePippin size selection step for subsequent analyses, which was able to fully reduce primer dimer presence (data not shown).

### 3.2 Quantitative PCR

**FIGURE 3:**
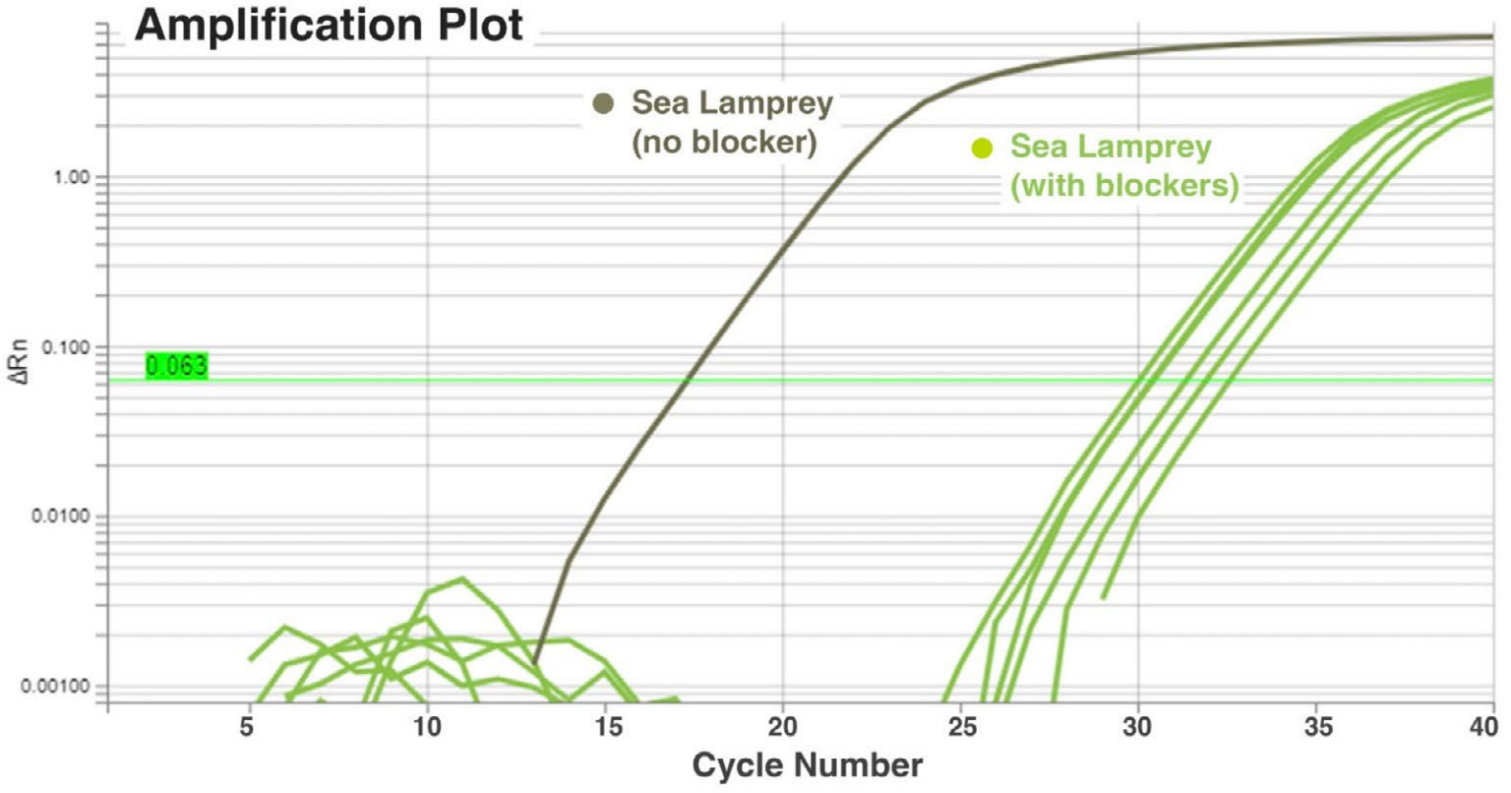
Amplification plot showing results for the quantitative polymerase chain reaction (qPCR) primer evaluation illustrating the cycle threshold (Ct) differences between blocker and no‐blocker reactions for sea lamprey DNA. Ct indicates the number of cycles it takes to reach the set value of 0.063 for the normalized change in fluorescence (ΔRn) as a proxy for the amplification of target DNA. The gray line represents one replicate of sea lamprey DNA with no‐blocking primers included in the reaction, and green lines represent the same sea lamprey DNA sample with each of the blocking primers included. The horizontal lime green line represents the threshold used to determine Ct values for each reaction.

Amplification plots were used to compare samples that only contained DNA from one species. Sea lamprey DNA amplification was compared in qPCR reactions with and without blocking primers, and the difference in Ct values of these reactions was used as a quantitative measure of the degree of amplification suppression (Figure 3). In sea lamprey samples that did not include a blocking primer, the mean Ct value across both replicates was 17.4 cycles (SD = 0.02). When blocking primers were included, the mean Ct value across replicates was 31.12 cycles (SD = 0.11), a mean Ct difference of 13.72 cycles or an average of 2^13.72^ = 13,494× suppression of sea lamprey amplification in reactions with a blocking primer. The best‐performing blocking primer in this comparison was Blocker 6 for both replicates (Ct values of 32.65 and 32.48 cycles). Single‐species samples of lake trout, walleye, and white sucker DNA were also compared between samples that did and did not contain blocking primers to assess inhibition of amplification of host species DNA. For lake trout, the mean Ct value difference between samples with and without a blocking primer was 1.28 cycles (SD = 0.17), indicating minimal suppression of 2.43×. Walleye samples had a mean Ct value difference of 3.37 cycles (SD = 0.22), resulting in a mean suppression of 10.3×. White sucker samples had the lowest mean Ct value difference at 0.59 cycles (SD = 0.11), an overall mean suppression of 1.5×. However, estimates of suppression may be conservative, as the presence of primer dimers in Figure 3 may have reduced our ability to accurately estimate levels of DNA suppression. In general, this is unlikely to influence the overall performance of blocking primers or the selection of best‐performing blocking primers.

**FIGURE 4:**
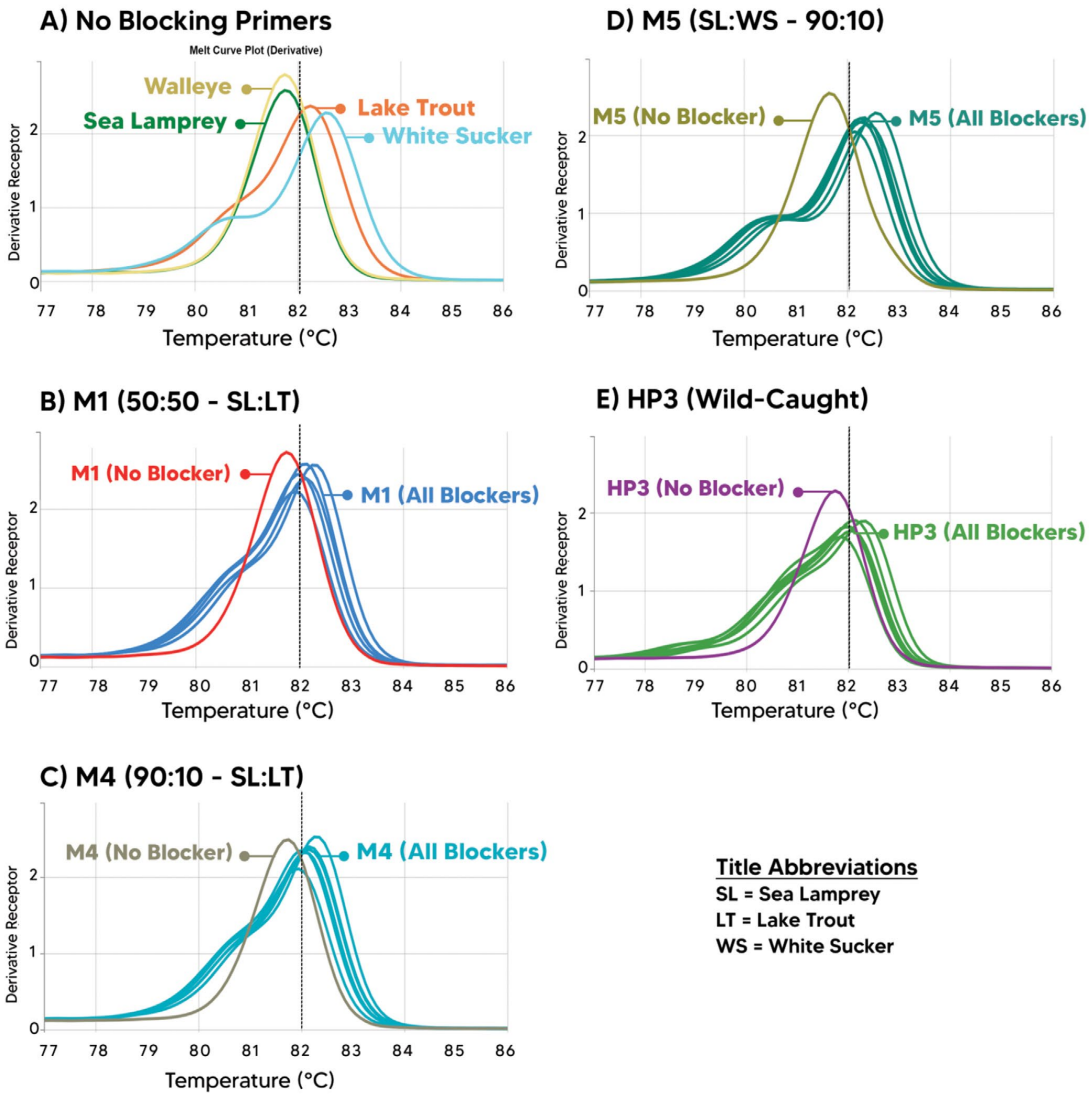
Melt curve plots showing results for the quantitative polymerase chain reaction (qPCR) primer evaluation method for M1 (1:1 sea lamprey:Lake trout DNA ratio), M4 (9:1 sea lamprey:Lake trout), M5 (9:1 sea lamprey:White sucker), and HP3 (wild‐caught Lake Huron juvenile sea lamprey) samples. (A) indicates the baseline melt curves for sea lamprey and host species amplicons (all single‐species samples with no‐blocking primer). (B–E) show the melt curves with and without a blocking primer to illustrate the shift in melting temperature (*T*_*m*_) across all blocking primers (blockers) that were included.

Melt curve plots were created (Figure 4) to examine the difference in melting temperature (*T*_*m*_) profiles between mixed‐species, host‐only, and sea lamprey‐only samples with and without blocking primers. Together, both sea lamprey sample replicates with no‐blocking primers generated a mean *T*_*m*_ of 81.77°C (SD = 0.03), establishing a sea lamprey baseline for the comparison of mixed‐species samples when a blocking primer was included. Similar baselines were established for host species (lake trout, white sucker, and walleye) to identify *T*_*m*_ shifts. For all four tested mixed samples, when a blocking primer was included, the *T*_*m*_ shifted from the sea lamprey baseline and toward the *T*_*m*_ of the associated host species. Additionally, all HP3 melt curves displayed a shift away from the sea lamprey baseline *T*_*m*_ and toward the lake trout baseline *T*_*m*_ with the inclusion of a blocking primer, indicating a higher presence of amplified lake trout DNA.

To visualize these shifts, melt curves displaying *T*_*m*_ peaks were plotted to demonstrate which DNA was being primarily amplified in mixed‐species samples when compared to the single‐species baselines (Figure 4). In all four mixed samples, the shape and peak of the melt curves for the unblocked sample closely resembled the melt curves for the sea lamprey sample. For all blocked samples, the shape and peak of the melt curves closely resembled that of the host species. Results indicate amplification of primarily sea lamprey DNA when no‐blocking primer was included, and amplification of primarily host fish DNA when a blocking primer was included for every mixed sample.

### 3.3 DNA Metabarcoding

**FIGURE 5:**
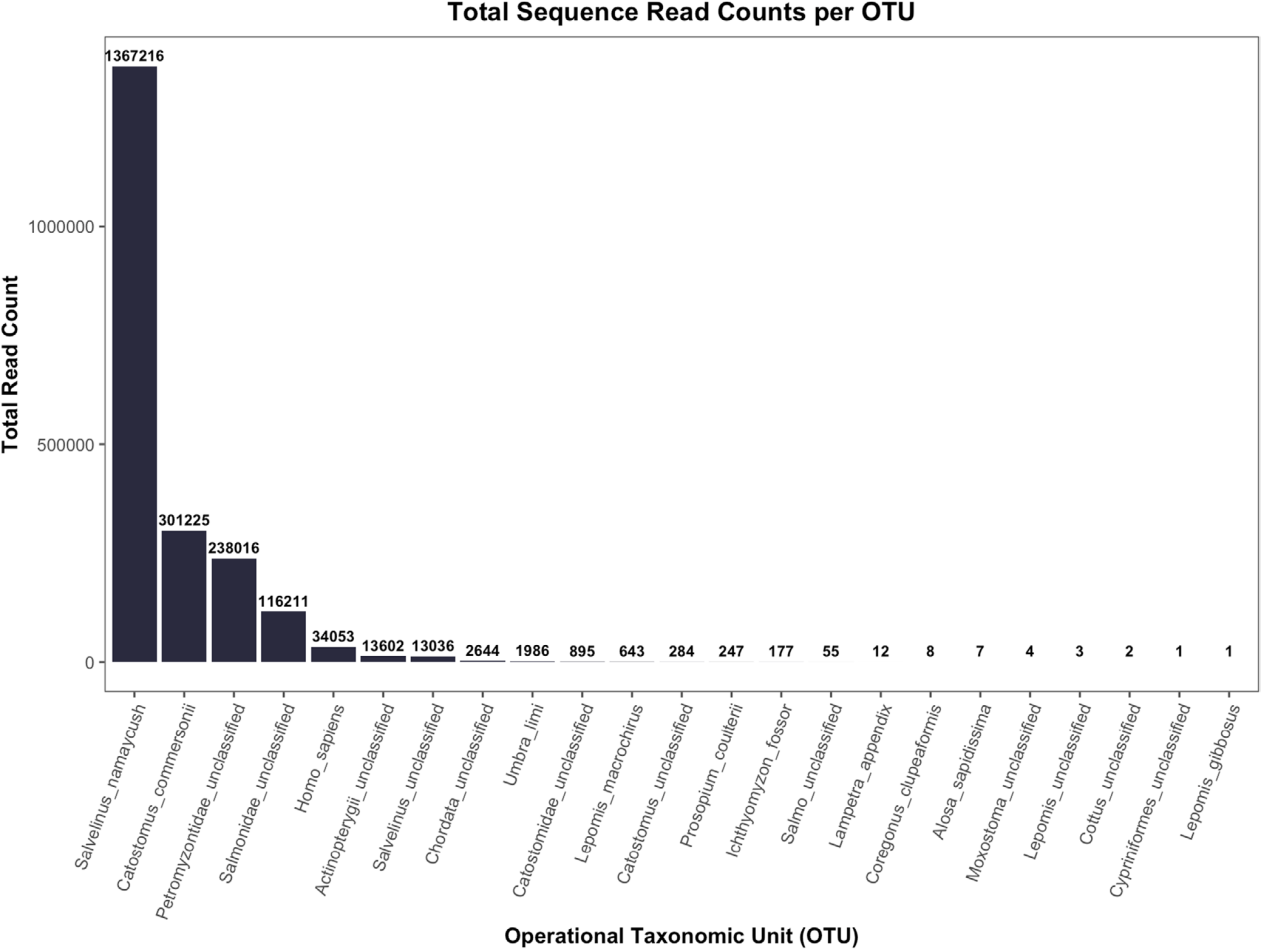
Total sequence read counts per operational taxonomic unit (OTU). For this study, only the top four OTUs were used, as they represented over 96% of the total sequence reads and included the known DNA from both mock community samples and single‐species samples. The additional OTUs were likely detections from the wild‐caught samples (HP3, HP5, HP15, and CA14) used in this study, as well as possibly being from contamination.

Initial analysis of DNA metabarcoding results via inspection of raw OTU sequence read counts from mothur showed a total of 23 OTUs with at least one sequence read. Of these 23 OTUs, the top four in terms of read count (lake trout, white sucker, Petromyzontidae, and Salmonidae) accounted for 96.76% of the total sequence reads (Figure 5). As the primary goal of this study was to compare the effectiveness of different blocking primers and not an in‐depth investigation into the dietary composition of wild‐caught sea lamprey, only these first four OTUs were used for blocking primer comparisons. Some OTUs with smaller proportional read counts could likely be grouped with these larger OTUs, such as categorizing *Salvelinus* unclassified as lake trout given the high quantities of known lake trout DNA in mock communities and observable attachment of wild‐caught sea lamprey‐to‐lake trout hosts. However, to avoid additional assumptions and given the low impact these groupings have on the results, none of the unclassified, lower‐proportioned OTUs were grouped with any of the top four OTUs. The only assumption made in terms of “unclassified” OTUs was that, given the prevalence of known sea lamprey DNA in both mock communities and wild‐caught samples, Petromyzontidae unclassified was considered to represent sea lamprey ( *Petromyzon marinus* ) reads. Assignments of sea lamprey sequences at the family level were expected, as the 12S sequence for sea lamprey in our reference database differs from three other lamprey species (American brook lamprey, *Lethenteron appendix* ; northern brook lamprey, *Ichthyomyzon fossor* ; and silver lamprey, *Ichthyomyzon unicuspis* ) by a single base pair.

**FIGURE 6:**
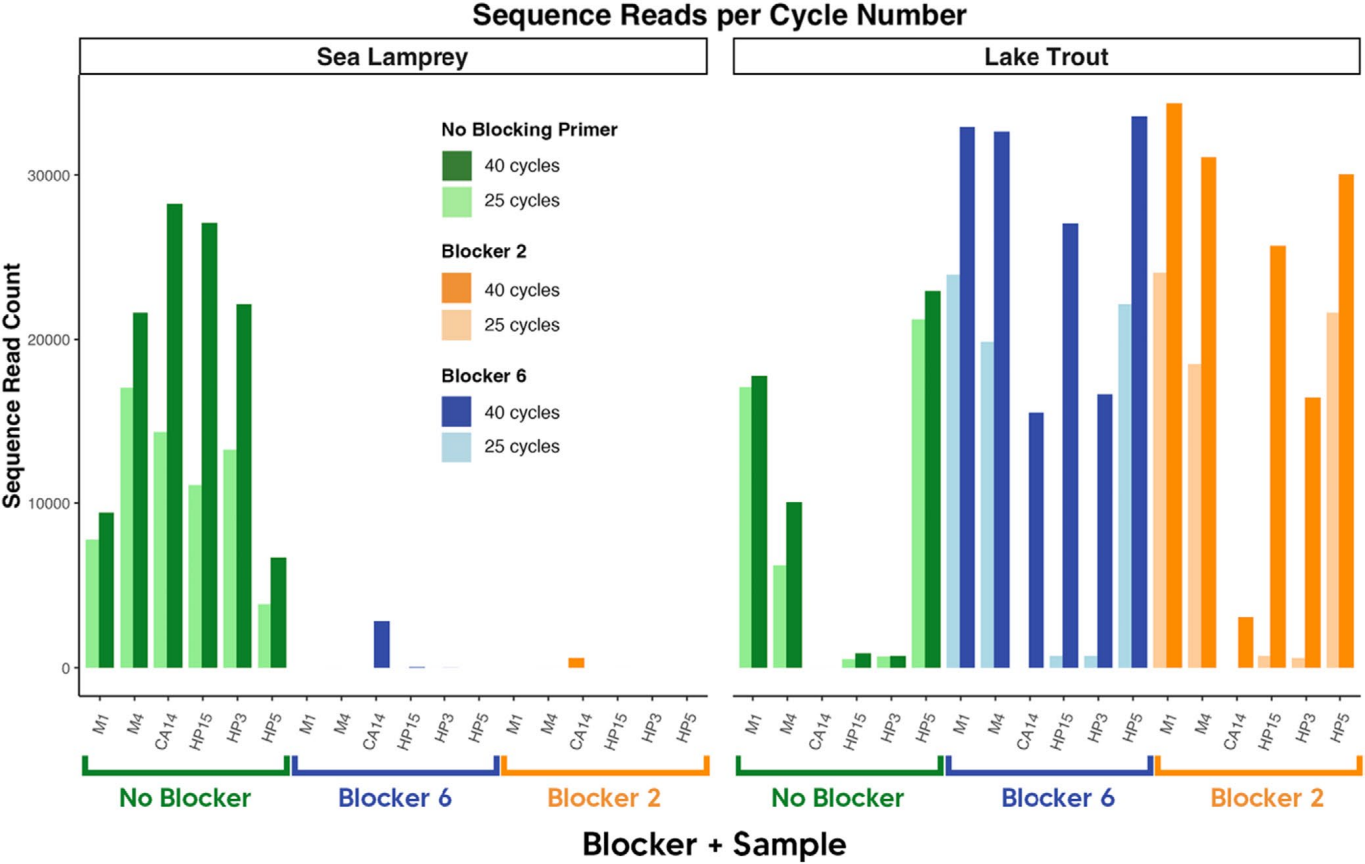
Sequence read counts for sea lamprey and lake trout from mixed‐species samples in 25 and 40‐cycle PCR runs with Blocker 2, Blocker 6, or no‐blocking primer included. Green columns indicate samples with no‐blocking primer, orange columns indicate Blocker 2 samples, and blue columns indicate Blocker 6 samples. Lake trout reads are consistently higher in 40‐cycle samples, with effective suppression of sea lamprey reads across both 25‐ and 40‐cycle samples.

Sequence read counts were visualized to compare differences between sea lamprey and lake trout community outputs based on 25‐cycle and 40‐cycle PCR runs (Figure 6). With sea lamprey, amplification suppression was high from both 25 and 40 cycles, indicating no benefit of cycle number. However, a noticeable improvement was made in the 40‐cycle PCR concerning the amount of host read amplification. In all samples, host read counts were higher from 40‐cycle PCR runs. In sample CA14, a wild‐caught adult sample from Lake Champlain, no lake trout reads were detected for either blocking primer in their respective 25‐cycle runs; however, both 40‐cycle runs with each blocking primer detected lake trout sequences. Sequence reads for the M5 sample with white sucker showed similar results.

**FIGURE 7:**
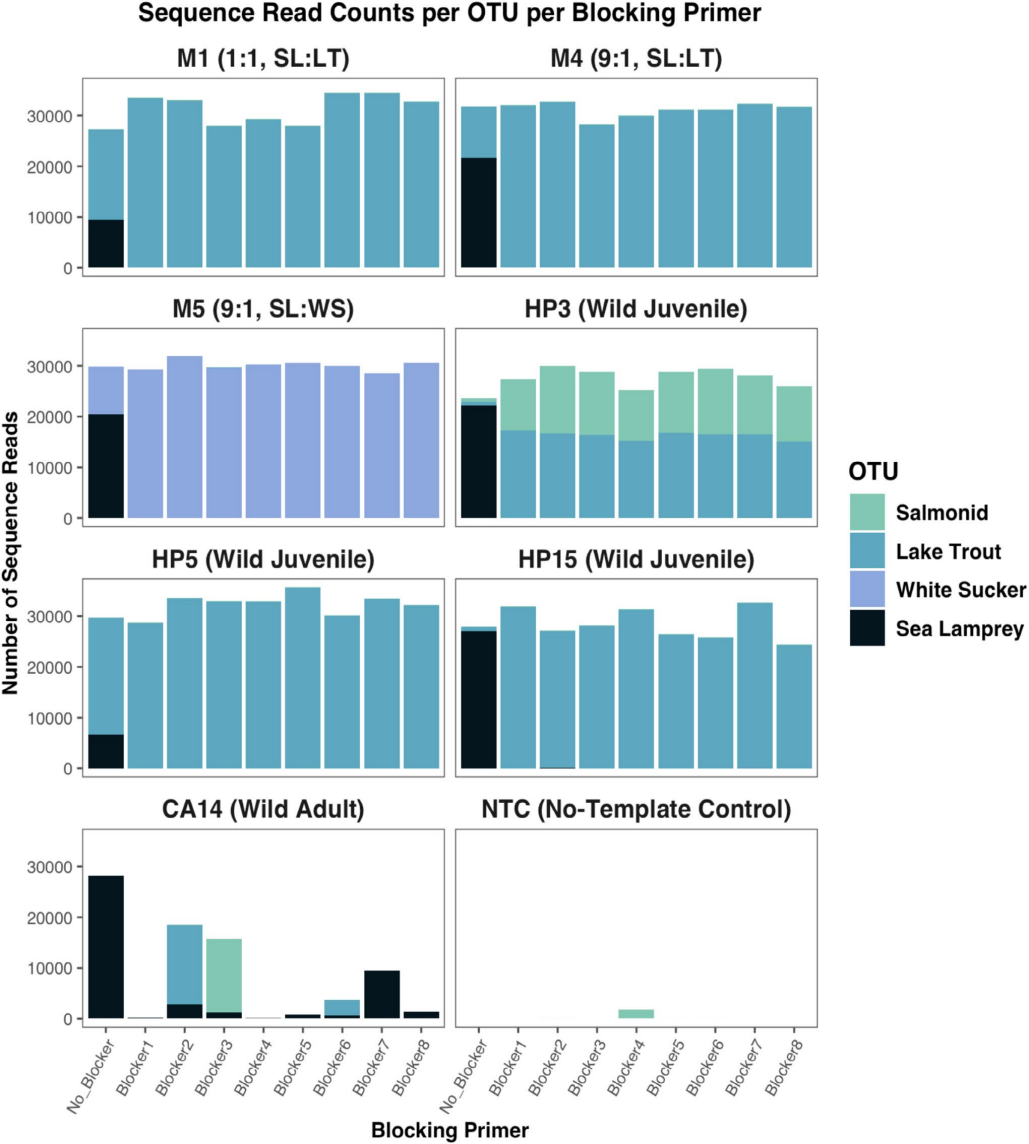
Sequence read count per operational taxonomic unit (OTU) comparisons between mixed‐species samples. The first column in each panel represents that sample with no‐blocking primer included. No‐template control (NTC) samples did not include DNA. High levels of host sequence amplification and lamprey sequence suppression are seen across all blocking primers, with CA14 providing insights into the most effective primers, as only Blockers 2, 3, and 6 allowed host detection.

All 40‐cycle samples were compared using the distribution of sequence reads per OTU with each blocking primer (Figure 7). A sample with no‐blocking primer (No_Blocker) was used as a baseline for comparisons. In all three mock communities (M1, M4, and M5), the number of sea lamprey reads with the inclusion of any blocking primer decreased an average of 99.99% across all mock communities, showing the efficacy of all tested blocking primers. Similarly, in Huron juvenile wild‐caught samples, even with high proportions of sea lamprey in samples without a blocking primer, all blocking primers were extremely effective in suppressing sea lamprey amplification (averaging a reduction of 99.98% of sequence reads) while still allowing for the amplification of host fish.

The only DNA sample to show clear discrepancies in blocking primer effectiveness was CA14, a wild‐caught spawning adult from Lake Champlain. Across all tested blocking primers, only three suppressed sea lamprey amplifications and allowed for host amplification in this sample. Of these three, Blockers 2 and 6 registered species‐level identification ( *Salvelinus namaycush* ) of the host, with Blocker 3 providing family‐level identifications (Salmonidae) of host sequences. Further investigation using BLAST with the NCBI GenBank database revealed that the salmonid reads were likely whitefish (*Coregonus* sp.) and unable to be distinguished between species within our reference database, thus resulting in family‐level classification. Given that no contamination of sequences was detected in the NTC samples for Blockers 2, 3, or 6, these host reads were designated as true positives. Overall, the proportion of host reads to total reads in these samples was higher with Blocker 3 included (91.7%) compared with Blockers 2 and 6 (84.6% and 83.2%, respectively).

Differences in read counts between reactions that included blocking primers and those that did not include blocking primers were evaluated using paired *t*‐tests (Table 3). All blocking primers significantly reduced sea lamprey read counts in dietary samples (*p <* 0.005), and significantly increased lake trout read counts (*p* < 0.05), with significance determined after a sequential Bonferroni correction. Additionally, *t* values were all > 6.0 for sea lamprey read count comparisons and all < −4.0 for lake trout read comparisons, emphasizing the effectiveness of all blocking primers. Full sequence read count data for both lake trout and sea lamprey for all samples (except M5 in lake trout comparisons) are included in Table 4.

**TABLE 3:** Paired *t*‐tests comparing read counts for samples that contain one of the eight blocking primers to samples that do not contain a blocking primer for both sea lamprey and lake trout.

Sea lamprey read count comparison			
Blocker	Mean (unblocked)	Mean (blocked)	*p*
Blocker 1	19387.29	35.57	0.0008
Blocker 2	19387.29	409.00	0.0007
Blocker 3	19387.29	187.71	0.0007
Blocker 4	19387.29	11.29	0.0008
Blocker 5	19387.29	114.43	0.0008
Blocker 6	19387.29	88.57	0.0008
Blocker 7	19387.29	1360.57	0.0006
Blocker 8	19387.29	194.71	0.0007

Lake trout read count comparison			
Blocker	Mean (unblocked)	Mean (blocked)	*p*
Blocker 1	8728	23866.83	0.0116
Blocker 2	8728	26386.50	0.0229
Blocker 3	8728	22226.17	0.0207
Blocker 4	8728	23057.67	0.0195
Blocker 5	8728	22948.50	0.0214
Blocker 6	8728	23439.50	0.0228
Blocker 7	8728	24838.50	0.0236
Blocker 8	8728	22606.00	0.0205

**TABLE 4:** Total sequence reads for lake trout (top) and sea lamprey (bottom) for all blocking primers and controls with each sample.

Lake trout sequence reads									
Sample	Unblocked	Blocker 1	Blocker 2	Blocker 3	Blocker 4	Blocker 5	Blocker 6	Blocker 7	Blocker 8
CA14	1	2	15,563	4	2	0	3063	1	0
HP15	891	31,764	27,026	28,104	31,264	26,392	25,667	32,604	24,249
HP3	715	17,312	16,679	16,338	15,124	16,742	16,468	16,457	15,026
HP5	22,915	28,638	33,531	32,896	32,817	35,572	30,021	33,338	32,155
M1	17,786	33,462	32,911	27,914	29,213	27,962	34,345	34,337	32,637
M4	10,060	32,023	32,609	28,101	29,926	31,023	31,073	32,294	31,569

Sea lamprey sequence reads									
Sample	Unblocked	Blocker 1	Blocker 2	Blocker 3	Blocker 4	Blocker 5	Blocker 6	Blocker 7	Blocker 8
CA14	28,238	246	2819	1305	76	785	617	9492	1352
HP15	27,068	0	37	1	1	3	1	15	2
HP3	22,131	2	7	4	0	10	0	10	7
HP5	6701	1	0	0	0	0	0	0	0
M1	9429	0	0	0	0	0	0	2	0
M4	21,633	0	0	4	2	3	1	1	2
M5	20,511	0	0	0	0	0	1	4	0

## 4 Discussion

### 4.1 Blocking Primer Effectiveness

In this study, we used three molecular interrogation methods (gel electrophoresis, quantitative PCR, and DNA metabarcoding) to evaluate the effectiveness of blocking primers for isolating and identifying prey species from sea lamprey digestive samples. All blocking primers tested were deemed effective in reducing the amplification of 12S mitochondrial rDNA sea lamprey sequences while retaining amplification of prey sequences, with the exception of the wild‐caught adult sample where only three blocking primers appeared effective (Blockers 2, 3, and 6). These three blocking primers varied in their design, representing both primer lengths, purification methods, and end modifications. Differences among blocking primer design variables did not appear to demonstrably influence performance. Given that host DNA was present in the adult sample only when one of these blocking primers was included, future studies may look to employ one of these three candidates (Blockers 2, 3, or 6) in the interest of suppressing 12S sea lamprey DNA amplification. However, the widespread effectiveness across all blocking primers in this study, in addition to the caveat that our results would benefit from more than a single adult sample, indicates that any of the tested blocking primers would be effective.

DNA metabarcoding can be a powerful tool for dietary analysis, particularly in the case of sea lamprey where it could be used to overcome hurdles posed by traditional methods. Current approaches to monitoring damage inflicted by sea lamprey have limitations, including failure to capture deceased prey, relying on targeted host capture, and subjectivity in wound assessments that prevent a comprehensive understanding of sea lamprey diets and prey preference (Adams et al. 2021). DNA metabarcoding could address some of these limitations by providing time, location, and species‐specific dietary profiles. Results from this study lay the groundwork for future research that will provide more extensive insights into Great Lakes Sea lamprey diets and their impact on native fishes. Furthermore, the development and rigorous evaluation of multiple candidate blocking primers in this study would be beneficial in any molecular diet analysis project where prey species are taxonomically grouped with the focal predator.

The effectiveness of blocking primers to suppress amplification of a nontarget species has been investigated previously, including in the context of dietary composition studies (Leray et al. 2013; Pertoldi et al. 2021; Homma et al. 2022). While some studies present sample richness comparisons of blocking primers against other predator DNA‐inhibiting techniques such as peptide nucleic acid (PNA) clamps or restriction enzymes, these studies often lack comprehensive comparisons of blocking primer variables and efficiencies (Lefèvre et al. 2020; Taerum et al. 2020). Often, end modifications such as C3 spacers and dT inversions are not fully contrasted, and a single end modification is chosen for all included blockers within the study (Huggins et al. 2020; Rojahn et al. 2021). Previous studies also often lack other forms of comparison such as variation in purification methods or the inclusion of mock communities with known DNA concentrations of specific species (De Barba et al. 2014; Toju and Baba 2018; Pertoldi et al. 2021). In our study, conventional PCR and gel electrophoresis provided a rapid, but limited evaluation of the candidate blocking primers. Quantitative PCR was also rapid and allowed us to evaluate the degree of amplification in single‐species samples, but interpretations of melt curves for mixed‐template samples can be more subjective (particularly when predator and prey amplicons are more similar than in this study). Finally, DNA metabarcoding provided a substantially more detailed view of read count ratios between sea lamprey and host species in mock communities and wild‐caught samples.

While all blocking primers were shown to be successful in the DNA metabarcoding dataset, the CA14 wild‐caught adult sample allowed for a detailed investigation of the effectiveness of all eight blocking primers, where only three were able to amplify host sequences within the sample. This adult was captured during upstream spawning migration and thus was unlikely to have fed recently at the time of capture as the juvenile feeding stage of its life cycle had concluded (Beamish 1980). As such, it is likely that lower amounts of dietary DNA were present, resulting in the noticeably higher sea lamprey‐to‐host sequence read ratio in the unblocked sample. When blockers were then included in the CA14 sample, only three were able to generate host reads. Blocker 2 and Blocker 6 both contained a C3 spacer and had a length of 36 bp, with the only difference being purification methods. Alternatively, Blocker 3 had a length of 34 bp and incorporated a dT inversion, along with HPLC purification. A notable difference among these three blocking primers is the detection of lake trout with Blockers 2 and 6 and the detection of whitefish with Blocker 3. This may be due to the high ratio of sea lamprey DNA compared to host DNA in the sample, indicated by low host read counts in the sample without a blocking primer. Given the stochasticity of PCR, if DNA from one of these species were amplified in an earlier PCR cycle, the exponential increase of those amplicons throughout the process may create a disproportionate amount of those amplicons in the final PCR product pool. For the purposes of this study, this still implies proper functioning of the blocking primer in reducing sea lamprey amplification while maintaining the ability to amplify host species DNA. With the ratio of sea lamprey sequences to host sequences from these three blockers in the CA14 sample being fairly comparable, it is likely that any of these blocking primers will suffice for future studies that employ this tool for sea lamprey dietary analyses.

By including multiple methods of analysis in this study, stronger conclusions about the efficacy of these blocking primers could be drawn. Across all three approaches, we saw that the blocking primers developed in this study consistently reduced sea lamprey DNA amplification. While host DNA was detected via DNA metabarcoding in all but one sample prior to the incorporation of the blocking primer, host reads represented as little as 3% of the sequence data from a sample. Thus, the use of a blocking primer substantially increased the efficiency of the analysis and should allow more reliable detection of prey items from sea lamprey digestive samples. Additionally, observed read ratios in mock community samples without blocking primers aligned with expected input proportions, validating our use of mock community samples to assess the utility of these blocking primers. Consistent performance across all three evaluation methods reinforces the reliability of these blocking primers and bolsters their applicability for future dietary analyses in sea lamprey.

### 4.2 Broader Applications

This study drew from previous work by Johnson et al. (2021) and their research applying DNA metabarcoding to sea lamprey fecal samples, but differs in several notable areas. In particular, this study assessed the use of a universal 12S rRNA vertebrate primer. In Johnson et al. (2021), three separate markers were used for the Salmonidae, Cyprinidae, and Catostomidae families. While the specificity can reduce amplification of predator sequences, this ultimately reduces the capability to detect rare host species from other families. Successful amplification of host species with a universal vertebrate primer, as shown in this study, provides a basis for molecular diet analysis in larger, lake‐wide studies of sea lamprey dietary compositions with the ability to detect rare taxa or to quantify dietary compositional variability spatially or temporally across samples and studies. Another previous study on Arctic lamprey diets also used universal vertebrate primers (Shink et al. 2019), emphasizing the benefit of this approach for broader improvements to dietary assessment across lamprey species and ranges.

While sea lamprey are invasive within the Great Lakes, they are threatened by various environmental stressors and sociopolitical issues within their native range in the North Atlantic (Hume et al. 2021). Knowledge transfer from sea lamprey management research in the Great Lakes to sea lamprey conservation efforts in their native range has thus been identified as a promising tactic for improving conservation of threatened populations (Guo et al. 2017). As such, the blocking primers developed in this study have the potential to not only improve our understanding of sea lamprey diets in the Great Lakes, but can also provide critical information on trophic ecology in the native range. Additionally, the focus on sea lamprey control within the Great Lakes has shifted attention away from native lamprey species within the region and their conservation needs (Lucas et al. 2021). DNA metabarcoding offers a useful approach for dietary analyses not only for sea lamprey, but for other lamprey species (particularly other blood‐feeding lampreys, that is, *Ichthyomyzon, Mordacia*, and *Petromyzon*; Renaud and Cochran 2019). Alongside sea lamprey, the reference database used in this study also contained 12S rRNA gene sequences for three Great Lakes native lamprey: American brook lamprey, northern brook lamprey, and silver lamprey. Base pair differences at 12S between native lamprey and sea lamprey were minimal (see Data Accessibility Statement), with American brook lamprey having a single nucleotide difference and both other native lamprey having no differences within the blocking primer annealing region. This taxonomic similarity implies these blocking primers may also be applicable in dietary assessments for conservation purposes in other parasitic native lamprey species (i.e., silver and chestnut lamprey).

### 4.3 Implications and Future Directions

Beyond the effectiveness of the blocking primers tested here, results from our study have implications for the application of molecular diet analysis in sea lamprey. In particular, successful amplification of hosts in the CA14 sample supports the feasibility of sea lamprey dietary assessments from migrating adults. These fish are typically captured via traps and barriers, creating a nonselective collection method (Miehls et al. 2020). Implementing DNA metabarcoding to analyze the feeding patterns from adult lamprey captured this way bypasses previous biases from the capture of juvenile sea lamprey, where host fish must first be targeted, captured, and assessed for attached lamprey or wound markings from a previous lamprey attack. Additionally, this would allow for host fish who did not survive a lamprey attack to still be included in analyses, as the DNA from that fish may still be present within the lamprey digestive tract whether the host survived the attack or not.

While the selected mock communities and wild‐caught samples in this study facilitated the determination of an effective blocking primer, this study could have benefitted from diet compositional data from additional adult samples. Collecting a larger number of juvenile and adult sea lamprey would have allowed for a more robust characterization of the efficiency of the candidate blocking primers in natural samples and may have revealed differences in performance that were not detected in this study. Furthermore, data from additional adult samples would have further demonstrated the feasibility of large‐scale applications of metabarcoding for sea lamprey dietary analysis. From a metabarcoding perspective, juvenile lamprey that have been immediately removed from a captured host and frozen for further analysis may harbor proportionately larger amounts of DNA from that immediate host. Even with a molecular feeding history, hosts which the lamprey fed on previously may escape detection due to this imbalance. Adult samples, in contrast, would not suffer from this potential bias as physical removal from an immediate host is not required. While the adult sample in this study did assist with the verification of an effective blocking primer, it would be beneficial to examine blocking primer performance across multiple adult samples to confirm the applicability of this approach.

Additionally, it should be noted that the presence of primer dimers observed in Figure 2 suggests potential amplification artifacts that could influence qPCR efficiency calculations. While suppression of sea lamprey amplification was evident, the presence of primer dimers may have inflated qPCR efficiency values above 100%; potentially impacting overall suppression estimates. Future studies may benefit from optimizing PCR conditions to further minimize primer dimer formation, such as adjusting primer concentrations, annealing temperatures, or using dimer‐reducing additives (e.g., DMSO, betaine). For the purposes of this study, the consistency of our results across other testing methods in addition to qPCR emphasizes the ability of these blocking primers to effectively suppress sea lamprey amplicons, even though exact suppression calculations from qPCR may have been biased from primer dimer detections.

Results are encouraging for future research into sea lamprey dietary composition such as experimental analyses of sea lamprey in controlled settings and in situ investigations for compiling a more in‐depth look into sea lamprey feeding patterns across the Great Lakes. Nonetheless, additional logistical questions need to be addressed prior to broad application of the molecular diet analysis methods used here and in Johnson et al. (2021). Specifically, experimental studies that explore how environmental variables such as temperature and fasting period impact sequence read count are needed (O'Kane 2024). With a better understanding of the effects of these variables on sequence reads and host detections, DNA metabarcoding would be available to be applied throughout the Great Lakes to improve our understanding of prey preference, damage to important Great Lakes fisheries, and ultimately, sea lamprey control and improvements to restoration of lampreys worldwide.

## Author Contributions

**Conor O'Kane:** conceptualization (equal), data curation (equal), formal analysis (equal), methodology (equal), visualization (equal), writing – original draft (equal). **Nicholas S. Johnson:** funding acquisition (equal), project administration (equal), resources (equal), writing – review and editing (equal). **Kim T. Scribner:** conceptualization (equal), funding acquisition (equal), methodology (equal), project administration (equal), writing – review and editing (equal). **Weiming Li:** methodology (equal), project administration (equal), writing – review and editing (equal). **Jeannette Kanefsky:** conceptualization (equal), methodology (equal), resources (equal), supervision (equal), writing – review and editing (equal). **John D. Robinson:** conceptualization (equal), funding acquisition (equal), methodology (equal), project administration (equal), supervision (equal), writing – review and editing (equal).

## Conflicts of Interest

The authors declare no conflicts of interest.

## Data Availability

The sequence data analyzed for this study are available in the NCBI Sequence Read Archive (Accession: PRJNA1209968). Code, data analysis scripts, and reference database (FASTA) are also available for sequence demultiplexing and quality filtering (https://github.com/okaneco1/blockingprimers).
